# Crystal structure of 2-amino-1,3-di­bromo-6-oxo-5,6-di­hydro­pyrido[1,2-*a*]quinoxalin-11-ium bromide monohydrate

**DOI:** 10.1107/S2056989015018253

**Published:** 2015-10-17

**Authors:** Md. Serajul Haque Faizi, Yuliia Parashchenko

**Affiliations:** a Department of Chemistry, College of Science, Sultan Qaboos University, PO Box 36 Al-Khod 123, Muscat, Sultanate of , Oman; b National Taras Shevchenko University, Department of Chemistry, Volodymyrska str. 64, 01601 Kiev, Ukraine

**Keywords:** crystal structure, hydrogen bonding, quinoxaline derivatives, pyrido[1,2-*a*]quinoxalines

## Abstract

In the title hydrated salt, C_12_H_8_Br_2_N_3_O^+^·Br^−^·H_2_O, which was synthesized by the reaction of the pyridine derivative Schiff base *N* ^1^,*N* ^4^-bis­(pyridine-2-yl­methyl­ene)benzene-1,4-di­amine with bromine, the asymmetric unit contains a 2-amino-1,3-di­bromo-6-oxo-5,6-di­hydro­pyrido[1,2-*a*]quinoxalin-11-ium cation, with a protonated pyridine moiety, a bromide anion and a water mol­ecule of solvation. The cation is non-planar with the di­bromo-substituted benzene ring, forming dihedral angles of 24.3 (4) and 11.5 (4)° with the fused pyridine and pyrazine ring moieties, respectively. In the crystal, the cations are linked through a centrosymmetric hydrogen-bonded cyclic *R*_4_^2^(8) Br_2_(H_2_O)_2_ unit by N—H⋯Br, N—H⋯O and O—H⋯Br hydrogen bonds, forming one-dimensional ribbons extending along *b*, with the planes of the cations lying parallel to (100).

## Abstract 2

In the title compound, the 2-amino-1,3-di­bromo-6-oxo-5,6-di­hydro­pyrido[1,2-*a*]quinoxalin-11-ium cations are non-planar and are linked through centrosymmetric hydrogen-bonded cyclic Br_2_(H_2_O)_2_ anion–water units by N—H⋯Br, N—H⋯O and O—H⋯Br hydrogen bonds, forming one-dimensional ribbons, with the planes of the cations lying parallel to (100).

## Chemical context   

Quinoxaline and its derivatives are an important class of benzo-heterocycles (Kurasawa *et al.*, 1988 ▸; Cheeseman & Werstiuk, 1978 ▸), displaying a broad spectrum of biological activities (Seitz *et al.*, 2002 ▸; Toshima *et al.*, 2002 ▸) which have made them important structures in combinatorial drug-discovery literature (Wu & Ede, 2001 ▸; Lee *et al.*, 1997 ▸). These compounds have also found applications as dyes (Zaragoza *et al.*, 1999 ▸; Sonawane & Rangnekar, 2002 ▸) and building blocks in the synthesis of organic semiconductors (Katoh *et al.*, 2000 ▸; Dailey *et al.*, 2001 ▸) and they also serve as useful rigid subunits in macrocyclic receptors for mol­ecular recognition (Mizuno *et al.*, 2002 ▸) and chemically controllable switches (Elwahy, 2000 ▸). The present work is a part of an ongoing structural study of Schiff bases and their utilization in the synthesis of new organic and polynuclear coordination compounds (Faizi & Sen, 2014 ▸; Moroz *et al.*, 2012 ▸). We report here the synthesis and crystal structure of 2-amino-1,3-di­bromo-6-oxo-5,6-di­hydro­pyrido[1,2-*a*]quinoxalin-11-ium bromide monohydrate (refcode ADOQBM). Previously, we have reported new methods for the preparation of substituted quinoxaline derivatives together with their crystallographic characterization. However, there are very few reported structures of compounds similar to the title compound, one being the doubly protonated dibromide salt 2-aza­niumyl-3-bromo-6-oxo-5,6-di­hydro­pyrido[1,2-*a*]quinoxalin-11-ium dibromide (Faizi *et al.*, 2015 ▸).

The title singly protonated monobromide monohydrate salt, C_12_H_8_Br_2_N_3_O^+^·Br^−^·H_2_O, was synthesized from the reaction the pyridine derivative Schiff base *N*1,*N*4–bis­(pyridine-2-yl­methyl­ene)benzene-1,4-di­amine (BPYBD) with mol­ecular bromine. The cyclization occurs by oxidation of BPYBD, reduction of mol­ecular bromine and finally hydrolysis of the imine bond which creates the charge at the pyridine nitro­gen atom in the quinoxaline ring system. The structure is reported herein.
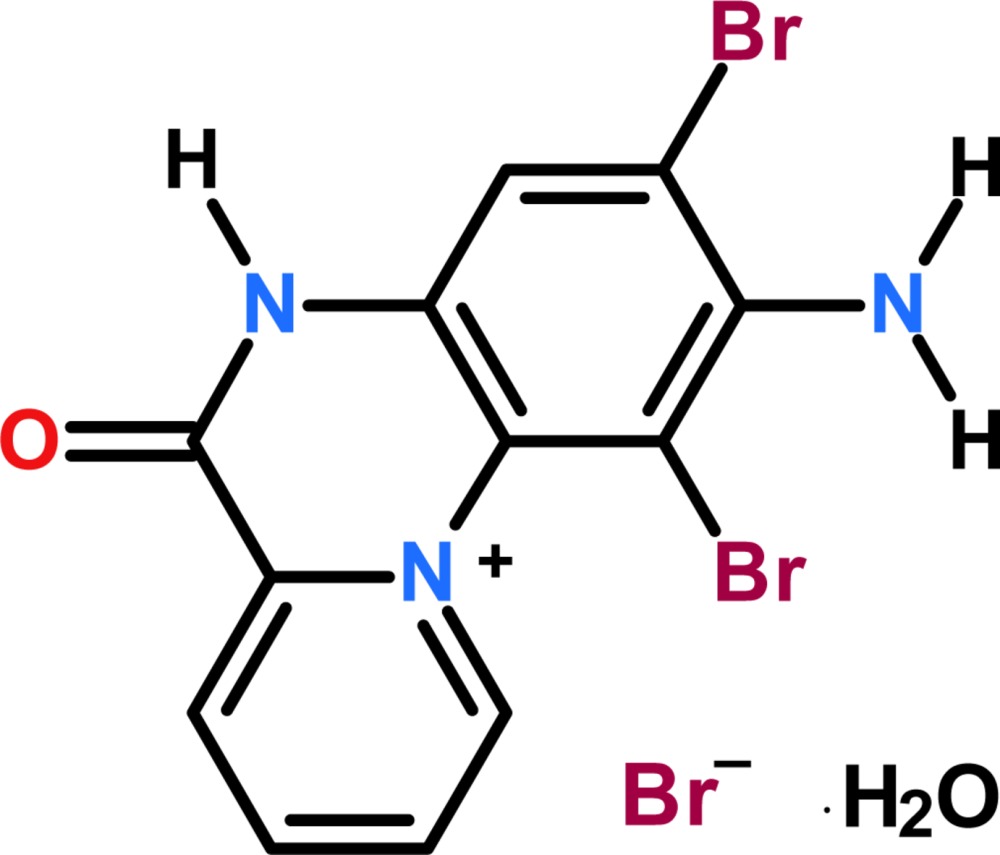


## Structural commentary   

**Figure 1:**
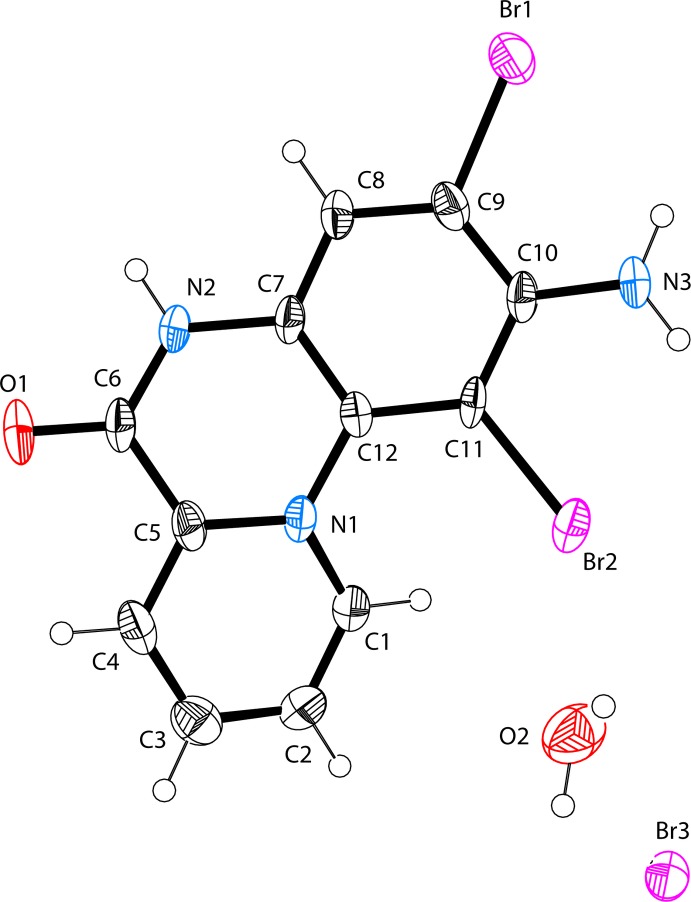
The mol­ecular conformation and atom-numbering scheme for the title compound, with non-H atoms drawn as 40% probability displacement ellipsoids.

The asymmetric unit of the title compound contains a discrete 2-amino-1,3-di­bromo-6-oxo-5,6-di­hydro­pyrido[1,2-*a*]quinoxalin-11-ium cation with a protonated pyridine moiety, and a bromide counter-anion and a water mol­ecule of solvation (Fig. 1 ▸). The cation is non-planar compared to the previously reported structure (Faizi *et al.*, 2015 ▸). The mean plane of the pyridine ring forms a dihedral angle of 24.2 (4)° with the benzene ring and 14.6 (4)° with the pyrazine ring of the fused system while the dihedral angle between the pyrazine and the benzene ring is 11.5 (4)°. A shorter C10—N3 distance of 1.367 (9) Å, compared to the usual aromatic C—N_amine_ single bond distance of 1.43 (3) Å, might be due to the electron-withdrawing effect of the positively charged pyridine N atom, and the *ortho*-substituted bromine atom which decreases the C—N_amine_ bond order. Other C—C and C—N bond distances are well within the limits expected for aromatic rings (Koner & Ray, 2008 ▸; Kanderal *et al.*, 2005 ▸; Fritsky *et al.*, 2006 ▸). Present also in the cations are intra­molecular N3—H⋯Br1 and N3—H⋯Br2 inter­actions [3.048 (7), 3.006 (7) Å, respectively, Table 1 ▸].

## Supra­molecular features   

**Figure 2:**
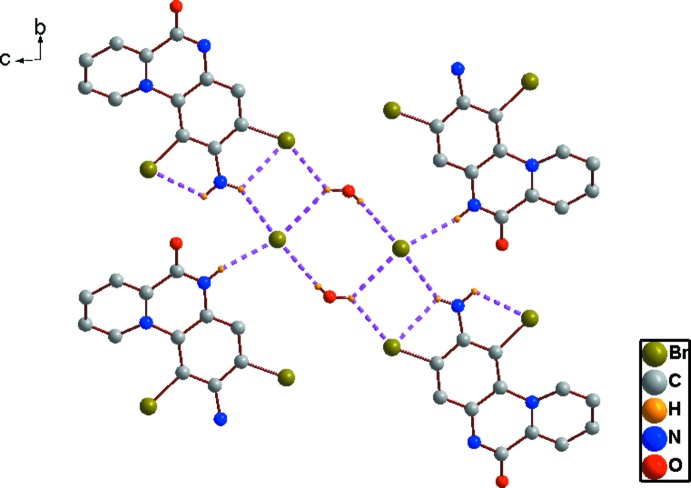
The one-dimensional hydrogen-bonded ribbon structure, viewed along the *a*-axis direction. Inter-species inter­actions are shown as dashed lines.

**Figure 3:**
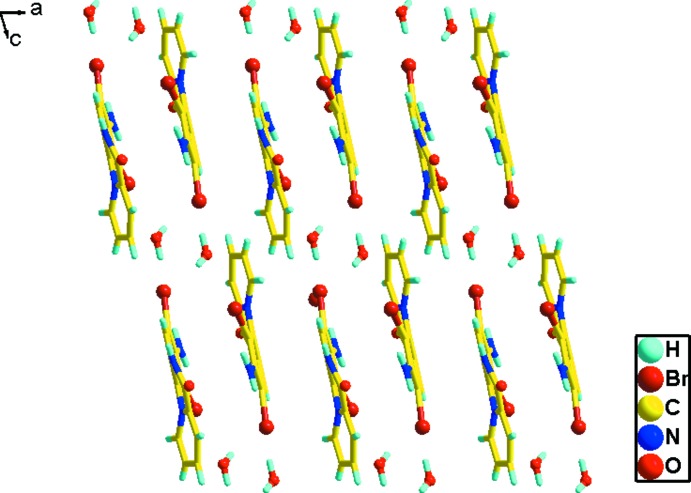
The layering of the ribbon structures, viewed along the *b* axis.

In the crystal, the cations are linked through a centrosymmetric hydrogen-bonded cyclic 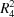
(8) Br_2_(H_2_O)_2_ unit and N—H⋯Br, N—H⋯O and O—H⋯Br hydrogen bonds (Table 1 ▸), forming broad one-dimensional ribbons extending along *b* (Fig. 2 ▸). The planes of the cations lie parallel to (100). Fig. 3 ▸ shows the packing in the unit cell, viewed along the *b* axis, in which layers of quinoxalinium cations are embedded between ionic layers of anions and *vice versa*, forming an alternating hydro­carbon–ionic layer structure. No inter­molecular π–π inter­ations are evident in the hydro­carbon layer in the structure.

## Database survey   

There are very few examples of similar compounds in the literature, a search of the Cambridge Structural Database (Version 5.35, May 2014 ▸; Groom & Allen, 2014) revealing the structure of 2-aza­niumyl-3-bromo-6-oxo-5,6-di­hydro­pyrido[1,2-*a*]quinoxalin-11-ium dibromide (Faizi *et al.*, 2015 ▸), in which the 2-amino-1,2-dibromide ring in the title compound is replaced by a 2-aza­niumyl-3-bromo ring. Other similar structures have been reported (Faizi & Sen, 2014 ▸; Koner *et al.*, 2008 ▸).

## Synthesis and crystallization   

Mol­ecular bromine (440 mg, 144.0 µL, 2.80 mmol) was added to a methano­lic solution (10 mL) of Schiff base, *N*1,*N*4-bis (pyridine-2-yl­methyl­ene)benzene-1,4-di­amine (BPYBD) (197 mg, 0.70 mmol). The color of the solution immediately changed from yellow to orange. The reaction mixture was stirred for 4 h at room temperature under a fume hood. The resulting yellow precipitate was recovered by filtration, washed several times with small portions of acetone and then with diethyl ether to give 200 mg (yield: 64%) of 2-amino-1,3-di­bromo-6-oxo-5,6-di­hydro­pyrido[1,2-*a*]quinoxalin-11-ium bromide monohydrate (ADOQBM). The crystal of the title compound suitable for X-ray analysis was obtained within three days by slow evaporation of a solution of the compound in methanol.

## Refinement   

Crystal data, data collection and structure refinement details are summarized in Table 2 ▸. All N-bound H atoms were located in difference-Fourier maps and their positions were then held fixed. The isotropic displacement parameters were refined for these atoms. Aromatic H atoms were placed in calculated positions and treated as riding on their parent C atoms [C—H = 0.93 Å and *U*_iso_(H) = 1.2 or 1.5*U*_eq_(C)].

## Supplementary Material

Crystal structure: contains datablock(s) global, I. DOI: 10.1107/S2056989015018253/zs2343sup1.cif

Structure factors: contains datablock(s) I. DOI: 10.1107/S2056989015018253/zs2343Isup2.hkl

CCDC reference: 1428593

Additional supporting information:  crystallographic information; 3D view; checkCIF report

## Figures and Tables

**Table 1:** Hydrogen-bond geometry (, )

*D*H*A*	*D*H	H*A*	*D* *A*	*D*H*A*
N2H5Br3^i^	0.86	2.49	3.332(6)	166
N3H3*B*Br1	0.86	2.60	3.048(7)	113
N3H3*B*Br3^ii^	0.86	2.84	3.581(7)	145
N3H3*A*O1^iii^	0.86	2.17	2.977(9)	155
N3H3*A*Br2	0.86	2.56	3.006(7)	113
O2H11Br3^iv^	0.89	2.50	3.383(6)	180
O2H12Br1^v^	0.88	2.61	3.309(7)	137

Symmetry codes: (i) 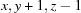; (ii) 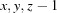; (iii) 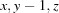; (iv) 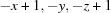; (v) 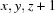.

**Table 2:** Experimental details

Crystal data	
Chemical formula	C_12_H_8_Br_2_N_3_O^+^BrH_2_O
*M*_r_	467.93
Crystal system, space group	Triclinic, *P* 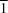
Temperature (K)	100
*a*, *b*, *c* ()	7.5069(7), 9.7435(10), 10.782(1)
, , ()	88.490(7), 73.798(7), 71.981(7)
*V* (^3^)	718.61(12)
*Z*	2
Radiation type	Mo *K*
(mm^1^)	8.42
Crystal size (mm)	0.20 0.15 0.11

Data collection	
Diffractometer	Bruker SMART APEX CCD
Absorption correction	Multi-scan (*SADABS*; Bruker, 2003 ▸)
*T*_min_, *T*_max_	0.259, 0.365
No. of measured, independent and observed [*I* > 2(*I*)] reflections	8077, 2187, 1681
*R*_int_	0.163
_max_ ()	23.8
(sin /)_max_ (^1^)	0.568

Refinement	
*R*[*F* ^2^ > 2(*F* ^2^)], *wR*(*F* ^2^), *S*	0.059, 0.155, 1.00
No. of reflections	2187
No. of parameters	181
H-atom treatment	H-atom parameters constrained
_max_, _min_ (e ^3^)	1.18, 1.16

Computer programs: *SMART* and *SAINT* (Bruker, 2003 ▸), *SIR97* (Altomare *et al.*, 1999 ▸), *SHELXL97* (Sheldrick, 2008 ▸) and *DIAMOND* (Brandenberg Putz, 2006 ▸).

## supplementary crystallographic information

### Crystal data

**Table:**

C_12_H_8_Br_2_N_3_O^+^·Br^−^·H_2_O	*Z* = 2
*M**_r_* = 467.93	*F*(000) = 448
Triclinic, *P*1	*D*_x_ = 2.163 Mg m^−^^3^
*a* = 7.5069 (7) Å	Mo *K*α radiation, λ = 0.71073 Å
*b* = 9.7435 (10) Å	Cell parameters from 1023 reflections
*c* = 10.782 (1) Å	θ = 1.5–23.5°
α = 88.490 (7)°	µ = 8.42 mm^−^^1^
β = 73.798 (7)°	*T* = 100 K
γ = 71.981 (7)°	Block, yellow
*V* = 718.61 (12) Å^3^	0.20 × 0.15 × 0.11 mm

### Data collection

**Table:**

Bruker SMART APEX CCD diffractometer	2187 independent reflections
Radiation source: fine-focus sealed tube	1681 reflections with *I* > 2σ(*I*)
Graphite monochromator	*R*_int_ = 0.163
/w–scans	θ_max_ = 23.8°, θ_min_ = 2.0°
Absorption correction: multi-scan (*SADABS*; Bruker, 2003)	*h* = −8→8
*T*_min_ = 0.259, *T*_max_ = 0.365	*k* = −11→10
8077 measured reflections	*l* = −12→12

### Refinement

**Table:**

Refinement on *F*^2^	Primary atom site location: structure-invariant direct methods
Least-squares matrix: full	Secondary atom site location: difference Fourier map
*R*[*F*^2^ > 2σ(*F*^2^)] = 0.059	Hydrogen site location: inferred from neighbouring sites
*wR*(*F*^2^) = 0.155	H-atom parameters constrained
*S* = 1.00	*w* = 1/[σ^2^(*F*_o_^2^) + (0.0902*P*)^2^] where *P* = (*F*_o_^2^ + 2*F*_c_^2^)/3
2187 reflections	(Δ/σ)_max_ < 0.001
181 parameters	Δρ_max_ = 1.18 e Å^−^^3^
0 restraints	Δρ_min_ = −1.16 e Å^−^^3^

### Special details

**Table:**

Experimental. The OH H-atom was located in difference Fourier map and refined with with *U*_iso_(H) = 1.2*U*_eq_(O). The N- and C-bound H-atoms were positioned geometrically and refined using a riding model: N—H = 0.86 Å and C—H = 0.93 Å with *U*_iso_(H) = 1.2*U*_eq_(parent atom).
Geometry. All esds (except the esd in the dihedral angle between two l.s. planes) are estimated using the full covariance matrix. The cell esds are taken into account individually in the estimation of esds in distances, angles and torsion angles; correlations between esds in cell parameters are only used when they are defined by crystal symmetry. An approximate (isotropic) treatment of cell esds is used for estimating esds involving l.s. planes.
Refinement. Refinement of F^2^ against ALL reflections. The weighted R-factor wR and goodness of fit S are based on F^2^, conventional R-factors R are based on F, with F set to zero for negative F^2^. The threshold expression of F^2^ > 2sigma(F^2^) is used only for calculating R-factors(gt) etc. and is not relevant to the choice of reflections for refinement. R-factors based on F^2^ are statistically about twice as large as those based on F, and R- factors based on ALL data will be even larger.

### Fractional atomic coordinates and isotropic or equivalent isotropic displacement parameters (Å^2^)

**Table:**

	*x*	*y*	*z*	*U*_iso_*/*U*_eq_	
Br3	0.26076 (12)	0.03614 (8)	0.73364 (8)	0.0441 (3)	
Br2	0.27561 (13)	0.36785 (8)	0.22264 (8)	0.0446 (3)	
Br1	0.30008 (13)	0.45076 (9)	−0.29950 (8)	0.0480 (3)	
C3	0.1893 (12)	0.8452 (10)	0.4590 (8)	0.048 (2)	
H3	0.2041	0.8791	0.5342	0.058*	
C1	0.1158 (12)	0.6740 (8)	0.3479 (7)	0.0388 (19)	
H1	0.0617	0.5998	0.3495	0.047*	
N1	0.1941 (9)	0.7217 (6)	0.2309 (6)	0.0316 (14)	
C2	0.1146 (14)	0.7316 (9)	0.4612 (8)	0.048 (2)	
H2	0.0644	0.6953	0.5393	0.057*	
C4	0.2417 (12)	0.9077 (9)	0.3430 (8)	0.046 (2)	
H4	0.2805	0.9898	0.3414	0.055*	
C12	0.2134 (10)	0.6506 (7)	0.1102 (7)	0.0306 (17)	
N2	0.2314 (10)	0.8738 (6)	0.0112 (6)	0.0377 (16)	
H5	0.2190	0.9269	−0.0528	0.045*	
C7	0.2241 (11)	0.7334 (7)	0.0019 (7)	0.0327 (18)	
C11	0.2384 (11)	0.5009 (7)	0.0921 (7)	0.0318 (18)	
C9	0.2558 (11)	0.5314 (8)	−0.1296 (8)	0.0362 (19)	
C10	0.2586 (10)	0.4390 (7)	−0.0288 (7)	0.0324 (18)	
C8	0.2379 (11)	0.6744 (7)	−0.1175 (8)	0.0349 (18)	
H6	0.2351	0.7313	−0.1879	0.042*	
C5	0.2364 (11)	0.8482 (8)	0.2307 (8)	0.0353 (18)	
C6	0.2565 (12)	0.9308 (8)	0.1142 (8)	0.042 (2)	
O1	0.2841 (12)	1.0476 (6)	0.1172 (7)	0.071 (2)	
N3	0.2950 (11)	0.2940 (7)	−0.0508 (7)	0.0457 (18)	
H3A	0.3064	0.2371	0.0109	0.055*	
H3B	0.3066	0.2597	−0.1263	0.055*	
O2	0.3545 (11)	0.2190 (7)	0.4635 (6)	0.072 (2)	
H12	0.3415	0.2338	0.5459	0.108*	
H11	0.4550	0.1520	0.4120	0.108*	

### Atomic displacement parameters (Å^2^)

**Table:**

	*U*^11^	*U*^22^	*U*^33^	*U*^12^	*U*^13^	*U*^23^
Br3	0.0499 (5)	0.0360 (5)	0.0489 (5)	−0.0143 (4)	−0.0173 (4)	0.0057 (4)
Br2	0.0516 (6)	0.0273 (5)	0.0573 (6)	−0.0140 (4)	−0.0184 (4)	0.0127 (4)
Br1	0.0565 (6)	0.0389 (5)	0.0510 (6)	−0.0130 (4)	−0.0206 (4)	−0.0041 (4)
C3	0.044 (5)	0.051 (6)	0.051 (5)	−0.013 (4)	−0.017 (4)	−0.003 (4)
C1	0.046 (5)	0.029 (4)	0.043 (5)	−0.014 (4)	−0.012 (4)	0.004 (4)
N1	0.033 (3)	0.022 (3)	0.045 (4)	−0.012 (3)	−0.016 (3)	0.007 (3)
C2	0.061 (6)	0.042 (5)	0.040 (5)	−0.016 (5)	−0.017 (4)	0.012 (4)
C4	0.052 (5)	0.033 (4)	0.060 (6)	−0.018 (4)	−0.021 (4)	−0.006 (4)
C12	0.032 (4)	0.022 (4)	0.040 (4)	−0.011 (3)	−0.010 (3)	0.000 (3)
N2	0.052 (4)	0.018 (3)	0.044 (4)	−0.010 (3)	−0.016 (3)	0.006 (3)
C7	0.036 (4)	0.016 (4)	0.047 (5)	−0.007 (3)	−0.015 (4)	0.006 (3)
C11	0.033 (4)	0.017 (4)	0.051 (5)	−0.014 (3)	−0.014 (3)	0.009 (3)
C9	0.037 (4)	0.030 (4)	0.048 (5)	−0.013 (4)	−0.018 (4)	−0.004 (4)
C10	0.029 (4)	0.016 (4)	0.053 (5)	−0.005 (3)	−0.017 (4)	0.004 (4)
C8	0.038 (4)	0.020 (4)	0.045 (5)	−0.008 (3)	−0.009 (4)	0.002 (3)
C5	0.036 (4)	0.018 (4)	0.049 (5)	−0.002 (3)	−0.014 (4)	−0.004 (3)
C6	0.053 (5)	0.017 (4)	0.057 (5)	−0.013 (4)	−0.016 (4)	0.002 (4)
O1	0.115 (6)	0.030 (3)	0.086 (5)	−0.041 (4)	−0.038 (4)	0.007 (3)
N3	0.069 (5)	0.022 (3)	0.052 (4)	−0.019 (3)	−0.022 (4)	0.003 (3)
O2	0.077 (5)	0.063 (4)	0.064 (4)	−0.005 (4)	−0.020 (4)	0.015 (4)

### Geometric parameters (Å, º)

**Table:**

Br2—C11	1.907 (7)	N2—C6	1.338 (10)
Br1—C9	1.912 (8)	N2—C7	1.393 (9)
C3—C2	1.384 (12)	N2—H5	0.8600
C3—C4	1.387 (12)	C7—C8	1.388 (11)
C3—H3	0.9300	C11—C10	1.400 (11)
C1—C2	1.355 (11)	C9—C8	1.364 (10)
C1—N1	1.370 (10)	C9—C10	1.394 (11)
C1—H1	0.9300	C10—N3	1.367 (9)
N1—C5	1.364 (9)	C8—H6	0.9300
N1—C12	1.440 (9)	C5—C6	1.471 (11)
C2—H2	0.9300	C6—O1	1.222 (9)
C4—C5	1.373 (11)	N3—H3A	0.8600
C4—H4	0.9300	N3—H3B	0.8600
C12—C7	1.399 (10)	O2—H12	0.8769
C12—C11	1.423 (9)	O2—H11	0.8900
			
C2—C3—C4	119.0 (8)	C12—C7—N2	120.3 (7)
C2—C3—H3	120.5	C10—C11—C12	121.3 (7)
C4—C3—H3	120.5	C10—C11—Br2	115.3 (5)
C2—C1—N1	122.1 (8)	C12—C11—Br2	123.0 (6)
C2—C1—H1	119.0	C8—C9—C10	124.3 (7)
N1—C1—H1	119.0	C8—C9—Br1	117.2 (6)
C5—N1—C1	118.1 (6)	C10—C9—Br1	118.3 (5)
C5—N1—C12	119.7 (6)	N3—C10—C11	122.5 (7)
C1—N1—C12	121.9 (6)	N3—C10—C9	121.0 (7)
C1—C2—C3	118.9 (8)	C11—C10—C9	116.3 (6)
C1—C2—H2	120.5	C9—C8—C7	118.8 (7)
C3—C2—H2	120.5	C9—C8—H6	120.6
C5—C4—C3	119.9 (8)	C7—C8—H6	120.6
C5—C4—H4	120.1	N1—C5—C4	120.2 (7)
C3—C4—H4	120.1	N1—C5—C6	120.8 (7)
C7—C12—C11	118.4 (7)	C4—C5—C6	118.7 (7)
C7—C12—N1	116.8 (6)	O1—C6—N2	123.8 (8)
C11—C12—N1	124.6 (6)	O1—C6—C5	120.2 (8)
C6—N2—C7	123.7 (6)	N2—C6—C5	115.9 (7)
C6—N2—H5	118.2	C10—N3—H3A	120.0
C7—N2—H5	118.2	C10—N3—H3B	120.0
C8—C7—C12	120.5 (7)	H3A—N3—H3B	120.0
C8—C7—N2	119.1 (7)	H12—O2—H11	123.0
			
C2—C1—N1—C5	−13.0 (11)	Br2—C11—C10—C9	−172.4 (6)
C2—C1—N1—C12	173.4 (7)	C8—C9—C10—N3	−173.9 (8)
N1—C1—C2—C3	2.1 (12)	Br1—C9—C10—N3	1.0 (10)
C4—C3—C2—C1	7.5 (12)	C8—C9—C10—C11	1.0 (12)
C2—C3—C4—C5	−6.1 (13)	Br1—C9—C10—C11	175.9 (5)
C5—N1—C12—C7	−16.6 (10)	C10—C9—C8—C7	0.9 (12)
C1—N1—C12—C7	156.8 (7)	Br1—C9—C8—C7	−174.0 (6)
C5—N1—C12—C11	157.9 (7)	C12—C7—C8—C9	−4.9 (11)
C1—N1—C12—C11	−28.7 (11)	N2—C7—C8—C9	171.8 (7)
C11—C12—C7—C8	6.7 (11)	C1—N1—C5—C4	14.3 (10)
N1—C12—C7—C8	−178.4 (7)	C12—N1—C5—C4	−172.0 (7)
C11—C12—C7—N2	−169.9 (7)	C1—N1—C5—C6	−159.2 (7)
N1—C12—C7—N2	5.0 (11)	C12—N1—C5—C6	14.4 (10)
C6—N2—C7—C8	−166.8 (7)	C3—C4—C5—N1	−5.0 (12)
C6—N2—C7—C12	9.8 (12)	C3—C4—C5—C6	168.7 (8)
C7—C12—C11—C10	−4.7 (11)	C7—N2—C6—O1	171.7 (9)
N1—C12—C11—C10	−179.2 (7)	C7—N2—C6—C5	−12.2 (11)
C7—C12—C11—Br2	168.1 (6)	N1—C5—C6—O1	176.1 (8)
N1—C12—C11—Br2	−6.4 (11)	C4—C5—C6—O1	2.5 (12)
C12—C11—C10—N3	175.8 (7)	N1—C5—C6—N2	−0.1 (11)
Br2—C11—C10—N3	2.4 (10)	C4—C5—C6—N2	−173.8 (7)
C12—C11—C10—C9	0.9 (11)		

### Hydrogen-bond geometry (Å, º)

**Table:**

*D*—H···*A*	*D*—H	H···*A*	*D*···*A*	*D*—H···*A*
N2—H5···Br3^i^	0.86	2.49	3.332 (6)	166
N3—H3*B*···Br1	0.86	2.60	3.048 (7)	113
N3—H3*B*···Br3^ii^	0.86	2.84	3.581 (7)	145
N3—H3*A*···O1^iii^	0.86	2.17	2.977 (9)	155
N3—H3*A*···Br2	0.86	2.56	3.006 (7)	113
O2—H11···Br3^iv^	0.89	2.50	3.383 (6)	180
O2—H12···Br1^v^	0.88	2.61	3.309 (7)	137
O2—H12···Br3	0.88	2.83	3.393 (6)	123

Symmetry codes: (i) *x*, *y*+1, *z*−1; (ii) *x*, *y*, *z*−1; (iii) *x*, *y*−1, *z*; (iv) −*x*+1, −*y*, −*z*+1; (v) *x*, *y*, *z*+1.
